# Quantitative liver SPECT/CT is a novel tool to assess liver function, prognosis, and response to treatment in cirrhosis

**DOI:** 10.3389/fmed.2023.1118531

**Published:** 2023-03-22

**Authors:** Amritjyot Kaur, Nipun Verma, Baljinder Singh, Ajay Kumar, Sunita Kumari, Arka De, Ratti Ram Sharma, Virendra Singh

**Affiliations:** ^1^Department of Nuclear Medicine, Post Graduate Institute of Medical Education and Research, Chandigarh, India; ^2^Department of Hepatology, Post Graduate Institute of Medical Education and Research, Chandigarh, India; ^3^Department of Transfusion Medicine, Post Graduate Institute of Medical Education and Research, Chandigarh, India

**Keywords:** decompensated cirrhosis, imaging, SPECT, mortality, G-CSF

## Abstract

**Background:**

Functional liver reserve is an important determinant of survival in cirrhosis. The traditional indocyanine green test (ICG) is cumbersome. Hence, we developed and validated a novel liver imaging, a hybrid of SPECT and CT (Q-SPECT/CT), for evaluating disease severity, outcomes, and response to treatment in decompensated cirrhosis (DC).

**Methods:**

We recruited a cohort of DC patients at a tertiary institute between 2016–2019. First, we standardized the Q-SPECT/CT across a predefined range of volumes through phantom experiments. Then we performed clinical and laboratory evaluations, ICG test (retention at 15 min), and Q-SPECT/CT at baseline and 12 months of granulocyte colony-stimulating factor (G-CSF) and standard medical treatment (SMT).

**Results:**

In 109 DC patients, 87.1% males, aged 51 ± 10 years, MELD: 14 (7–21), the percent quantitative liver uptake (%QLU) on Q-SPECT/CT exhibited a strong correlation with CTP (*r* = −0.728, *p* < 0.001), MELD (*r* = −0.743; p < 0.001) and ICG-R-15 (*r* = −0.720, p < 0.001) at baseline. %QLU had the maximum discrimination (AUC: 0.890–0.920), sensitivity (88.9–90.3%), specificity (81.2–90.7%), and accuracy (85.8–89.4%) than liver volumes on Q-SPECT/CT or ICG test for classifying patients in CTP/MELD based prognostic categories. A significant increase in %QLU (26.09 ± 10.06 to 31.2 ± 12.19, *p* = 0.001) and improvement in CTP/MELD correlated with better survival of G-CSF treated DC patients (*p* < 0.05). SMT did not show any improvement in Q-SPECT/CT or clinical severity scores (*p* > 0.05). %QLU > 25 (adj.H.R.: 0.234, *p* = 0.003) and G-CSF treatment (adj.H.R.: 0.414, *p* = 0.009) were independent predictors of better 12-months survival in DC.

**Conclusion:**

Q-SPECT/CT (%QLU) is a novel non-invasive, diagnostic, prognostic, and theragnostic marker of liver reserve and its functions in cirrhosis patients.

**Clinical trial registration:**

Clinicaltrials.gov, NCT02451033 and NCT03415698.

## 1. Introduction

Decompensated cirrhosis (DC) and associated complications such as ascites, gastrointestinal bleeding, infections, hepatic encephalopathy, and hepatocellular carcinoma portend high morbidity and mortality (1, 2). Child-Turcotte-Pugh (CTP) and Model for end-stage-liver disease (MELD) score traditionally assess liver functions and prognosticate cirrhosis patients. However, these scores have inherent limitations for the precise evaluation of liver functions and assessment of response to therapies (3, 4). Problems with CTP score include subjective assessment of ascites and encephalopathy, overestimation of severity due to increased bilirubin in cases of renal insufficiency, hemolysis, and decreased prothrombin complex due to activation of coagulation in case of sepsis (5, 6). All such factors contribute to over or underestimation of the severity of the liver disease. MELD is commonly used for prognosticating and prioritizing patients waiting for liver transplantation (7). It is a validated marker for predicting survival; however, its utility is often impeded due to lab-to-lab variations and different methods used for estimating the creatinine and INR values (8).

Although the indocyanine green (ICG) clearance test is a reference standard for the quantitative assessment of liver functions, it is cumbersome, requires multiple blood samplings, and has a high blood flow dependency. Thus, it is not used in routine clinical practice (9–15). A high functional heterogeneity among the hepatic segments is observed in liver cirrhosis and portal vein thrombus, which may lead to erroneous measurements. We hypothesize that non-invasive and quantitative imaging that can depict the spatial and segmental distribution of the radiotracer in the liver may become a potential substitute for ICG (16, 17). Sulfur-colloid (SC), which is extracted by Kupffer cells with no further clearance, is a surrogate molecule to estimate hepatocyte functions which can be assessed semi-quantitatively by single photon emission computed tomography (SPECT) (16, 18). Further, with the recent advances in image processing, the accurate quantification of the radioactivity distribution is possible. A hybrid SPECT/CT imaging can improve the diagnostic performance for the *in vivo* characterization of liver functions in cirrhosis (19–21).

Granulocyte colony-stimulating factor (G-CSF) is an emerging regenerative treatment in DC G-CSF mobilizes bone marrow stem cells (CD34+ cells) in peripheral blood, which can populate the liver tissue and differentiate into hepatic cells (22–24). Although a complete understanding of G-CSF action is unclear in cirrhosis, the safety and efficacy of G-CSF in hepatitis and chronic liver diseases have been reported (22, 24–26).

In this study, we examined the utility of novel non-invasive quantitative ^99m^Tc-SC liver SPECT/CT for assessing disease severity, patient outcomes, and therapeutic response to G-CSF treatment in DC patients.

## 2. Materials and methods

### 2.1. Patients

We recruited 109 DC patients (95Males:14Females; mean age-51 ± 10 years) randomly assigned to either G-CSF + SMT or SMT (standard medical therapy) in the ongoing studies (25, 26) at a tertiary care center between January 2016 to March 2019. Patient selection criteria and study protocols (NCT02451033, NCT03415698) have been published previously (Supplementary Figures S1, S2).

Briefly, we included DC patients aged 18–80 years with a history of ascites and/or variceal bleeding and/or encephalopathy and/or jaundice. The cirrhosis was diagnosed through the clinical presentation, biochemical tests, radiology, endoscopy, and/or histopathology. We excluded patients with acute-on-chronic liver failure, sepsis, recent variceal bleed, grade III-IV hepatic encephalopathy, acute kidney injury, hepatocellular carcinoma or active malignancy, spleen>18 cm, portal vein thrombosis, cardiac dysfunction, recent alcohol abuse in 3 months, or alcoholic hepatitis, seropositivity for H.I.V., pregnancy, and hypersensitivity to G-CSF. We obtained written informed consent from all participants, followed GCP guidelines, and the protocol was approved by the Institute Ethics Committee (N.K./2,722/Ph.D./1,224).

The eligible patients were randomized using a computer-generated random number table with allocation concealment in the two groups (Supplementary Figure S2). The patients (n = 68) of group-A received standard medical therapy (SMT) plus G-CSF treatment at a dose rate of 5.0 μg/kg subcutaneously every 12.0 h for five consecutive days. This group received four similar cycles of G-CSF treatments at 3.0 months intervals over 1.0 years. The patients (n = 41) of group B received only standard medical therapy (SMT), which included: nutritional support, rifaximin, lactulose, bowel wash, albumin, diuretics, multivitamins, and antibiotics as per guidelines. Fresh frozen plasma and packed red-cell transfusions were administered as and when indicated. Stem cell mobilization was tested with CD34 cell estimation in peripheral blood at baseline and 6 days after therapy in both groups. The patients were followed up till 12 months, death or transplant (whichever was earlier) with physical examination, biochemical parameters, and CTP/MELD scoring. The patients were classified on CTP and MELD-based prognostic categories as CTP-A (n = 10), CTP-B (n = 56), and CTP-C (n = 43) and MELD of ≤15.0 (n = 77) and > 15.0 (n = 32). Study subjects underwent ^99m^Tc-SC liver and spleen SPECT and ICG tests at baseline and follow-up. The Q-SPECT parameters were evaluated and correlated with the CTP/MELD score and ICG-R15 in both groups of patients.

### 2.2. Phantom standardization of Q-SPECT/CT

A series of phantom (Jaszczak SPECT phantom, Biodex Medical Systems, Inc., NY, United States) studies were performed using varying volumes (6.0 mL to 3,200 mL) and different radioactivity concentrations (28.5 kBq/mL to 151.33 kBq/mL) of pertechnetate (^99m^TcO_4_^−^) to develop and standardize the optimal processing protocol for accurate quantification (Supplementary Figures S3–S6; Supplementary Table S1). Data were acquired on a dual-headed gamma camera (Symbia- T16, SIEMENS, Erlangen, Germany) in 120 projections (20s/projection) in a 128 × 128 matrix with a zoom factor of 1.0. The data acquired was reconstructed by iterative reconstruction using a butter worth filter (cut off 0.5 cycles/cm) on a dual-head gamma camera. The threshold values varied from 19.0 to 52.0 to obtain the best volume estimation. Counts/voxel were converted into concentration units (μCi/mL) using the regression line, which was used to calculate liver/spleen volume, quantitative liver /spleen uptake, the percentage of injected dose per milliliter of liver/spleen tissue (%ID/mL) respectively.

### 2.3. Patients’ imaging with ^99m^Tc-S-colloid liver SPECT

Briefly, 4.0–5.0 mCi (148- to 185-MBq) radioactivity of ^99m^Tc-SC was injected intravenously in each patient (Supplementary Figure S7). Planar liver imaging was done at 20-min post-injection, followed by SPECT/CT acquisition at 30-min using the same gamma camera described above for the phantom standardization. The camera peaked at 140.0 keV with a window of ±20.0%. SPECT data were acquired over 360-degree rotation in 120 projections (20 s/ projection) in a 128 × 128 matrix (zoom, 1.5). After SPECT acquisition, low-dose CT (60 mAs and 140 kVP) was performed for the liver region. The images were reconstructed using an iterative reconstruction algorithm and Butterworth smoothing filter. The reconstructed images were analyzed using 3D volumetric analysis software 8.5.10.1 (Symbia.net, Munich, Germany). R.O.I.s were drawn coronal slices using a fixed threshold iso-contouring method to obtain volume and radioactive concentration.

### 2.4. Indocyanine green retention test

ICG (0.3 mg/kg of body weight) was administered intravenously *via* a peripheral vein. Blood samples were collected at 0, 5-, 10-, 15-, and 20-min post-injection from the contralateral vein. A standard dilution curve was obtained by using varying ICG concentrations. The same was used to estimate the unknown blood ICG levels to measure the dye retention in the liver. The ICG concentrations in standard solution and blood were recorded at 805 nm using a spectrophotometer (Systronics, UV/VIS spectrophotometer118, Gujarat, India). Retention value and plasma disappearance rate were calculated for all the patients. The standard reference ICG retention value was taken as less than 10.0% at 15-min and plasma disappearance rate of more than 18.0 mL/min.

### 2.5. Fluorescence-activated cell sorting analysis

Fluorescence-activated cell sorting (FACS) technique was used to estimate the circulating hematopoietic progenitor cells (CD34+) in peripheral venous blood on day 0 and at day 6 of initiation of G-CSF therapy using a flow cytometer (BD FACS Canto II, San Jose, California, U.S.A.) (Supplementary Figures S8, S9). Precisely, 2.0 mL of blood was collected in EDTA vacutainer for total nucleated cell count and CD34+ cells estimation.

### 2.6. Statistical analysis

Numerical values were expressed as mean ± SD or median (range) as appropriate. The unpaired t-test or chi-square was used to compare numerical and categorical variables between groups. A paired t-test or u-test was used to compare the pre-and post-treatment numerical parameters. ANOVA was used to compare numerical data between the three groups. The correlation between numerical variables was expressed through the Pearson correlation coefficient (*r*) at 95% confidence intervals. Receiver operating characteristics (R.O.C.) curves were used to calculate the area under the curve (AUC), sensitivity, specificity, positive and negative predictive values, and accuracy for differentiating severity grades in liver cirrhosis. The optimal cut-offs of ICG and Q-SPECT/CT for defining severity and mortality were obtained from maximum Youden’s index. Kaplan Meier survival and cox-regression were conducted to evaluate survival estimates and hazards of mortality between groups, and the groups were compared with the Log-Rank test. Transplanted and lost to follow-up patients were considered an event for a 12-month transplant-free survival analysis. All statistical tests were two-sided and performed using IBM SPSS v.22 and RStudio v.1.4.1103 at a significance level of *p* < 0.05.

## 3. Results

We recruited patients aged 51.1 ± 9.3 years, 86% males, predominantly alcohol-related cirrhosis (54.5%), with a CTP of 9 (6–13) and MELD of 14 (7–21) (Supplementary Table S2).

### 3.1. Standardization protocol

The best threshold value of Q-SPECT was calculated from the phantom imaging data using 3D volumetric analysis (Supplementary Figures S5, S6; Supplementary Table S1). These values were found to be 41.0, 38.0, and 33.0 for activity volume ranges of 6.0–30.0 mL, 500.0–1500.0 mL, and 2,400–3,200 mL, respectively. The estimated threshold values provided significant (*p* = 0.001) correlation of 0.90, 0.98 & 0.97 for the three reference ranges. Further, by using the threshold of 38.0%, the SPECT reconstructed data was used to calculate the different range (28.5 kBq/mL to 151.33 kBq/mL) of radioactivity concentrations and compared with the actual concentrations used for this standardization procedure. For calculating the tracer radioactivity, the regression line equation on attenuation corrected images used was: counts/cc = 6807.3 × μci/cc + 39.0. We used the best-fitted regression line to calculate the volume and radioactivity concentration at baseline and post-G-CSF treatment SPECT data in all the patients (Supplementary data).

### 3.2. Descriptive Q-SPECT and ICG-test in cirrhosis

The Q-SPECT and ICG parameters differed in CTP and MELD-based prognostic categories, as illustrated in Table 1. The liver volumes (LV) observed in CTP-C patients were significantly lower than that in CTP-B and A patients (*p* = 0.028) (Supplementary Figure S8A). The fractional liver uptake (%QLU) was substantially lower in CTP-C patients than in CTP-B and A (*p* = 0.001) (Supplementary Figure S8B). Likewise, liver volumes and %QLU were lower in the MELD>15 groups than MELD≤15 group, with *p* = 0.003 and *p* < 0.001 (Supplementary Figures S9A,B). Quantitative spleen parameters were not different between various Child and MELD classes (*p* > 0.05) (Table 1). ICG-R15 was significantly lower in CTP-A and B than in C and MELD≤15 than >15 groups (*p* < 0.001) (Supplementary Figure S10).

**Table 1:** Q-SPECT/CT and ICG parameters among prognostic categories in decompensated cirrhosis.

Parameters	CTP category				MELD category		
	CTP-A (*n* = 10)	CTP-B (*n* = 56)	CTP-C (*n* = 43)	*p*-Value	MELD<15 (*n* = 77)	MELD>15 (*n* = 32)	*p*-Value
*Q-SPECT/CT: Liver parameters*							
LV (mL)	1,200 ± 356	994 ± 253	860 ± 250	0.028	1,021 ± 288	819 ± 205	0.003
%QLU	41.20 ± 3.77	29.79 ± 6.93	15.9 ± 7.83	0.001	30.1 ± 9.0	15.25 ± 5.80	<0.001
%ID/mL (Liver)	0.038 ± 0.016	0.031 ± 0.009	0.019 ± 0.009	0.835	0.031 ± 0.012	0.019 ± 0.007	<0.001
*Q-SPECT/CT: Spleen parameters*							
SV (mL)	717 ± 457	644 ± 380	735 ± 303	0.997	661 ± 378	734 ± 319	0.264
%QSU	28.10 ± 13.73	29.79 ± 13.41	34.57 ± 12.83	0.940	29.14 ± 12.64	35.36 ± 14.47	0.085
%ID/mL (Spleen)	0.045 ± 0.022	0.051 ± 0.018	0.052 ± 0.021	0.321	0.050 ± 0.019	0.053 ± 0.019	0.988
*ICG-test*							
R-15 (%)	35.22 ± 8.33	44.4 ± 10.50	54.10 ± 12.03	<0.001	44.62 ± 12.08	53.97 ± 10.71	<0.001

Q-SPECT/CT, Quantitative single photon emission computed tomography; ICG, Indocyanine green; CTP, Child-Turcotte-Pugh; MELD: Model for end stage liver disease; LV, Liver Volume; %QLU, Percentage Quantitative Liver Uptake; %ID/mL, Injected Dose per milliliter; SV, Spleen Volume; %QSU, percentage Quantitative Spleen Uptake; ICG-15, Indocyanine green test (retention value at 15 min).

### 3.3. Correlation of Q-SPECT with cirrhosis severity and ICG-test

Table 2: The %QLU exhibited a strong inverse correlation with CTP (*r* = −0.728, *p* < 0.001), MELD (*r* = −0.743; p < 0.001) and reference standard ICG-R15 (*r* = −0.720, *p* < 0.001) values, respectively. The percentage of injected dose/mL also showed a significant but moderate correlation with CTP, MELD, and ICG-R15, followed by LV. However, no significant correlation was observed for CTP and MELD scoring with any quantitative spleen parameters. Analysis between CTP, MELD, and ICG parameters (ICG R-15 and P.D.R.) also demonstrated a negative correlation between CTP *and* ICG R-15 (*r* = −0.670; *p* = <0.001); CTP *and* P.D.R. (*r* = −0.670; *p* = <0.001); MELD and ICG R-15 (*r* = −0.521; *p* = <0.001), MELD *and* P.D.R. (*r* = −0.550; *p* = <0.001), respectively.

**Table 2:** Correlation# between quantitative Q-SPECT/CT and CTP, MELD scores, ICG parameters.

99mTc-SC SPECT parameters	CTP	MELD	ICG R-15	PDR
LV	*r* = − 0.409	*r* = −0.370	*r* = −0.427	*r* = 0.450
	*p* < 0.001	*p* < 0.001	*p* < 0.001	*p* < 0.001
%QLU	*r* = −0.728	*r* = −0.743	*r* = −0.720	*r* = 0.754
	*p* < 0.001	*p* < 0.001	*p* < 0.001	*p* < 0.001
% ID/mL liver	*r* = −0.471	*r* = −0.538	*r* = −0.453	*r* = 0.461
	*p* < 0.001	*p* < 0.001	*p* < 0.001	*p* < 0.001
SV	*r* = 0.089	*r* = 0.124	*r* = 0.157	*r* = −0.176
	(n.s.)	(n.s.)	(n.s.)	(n.s.)
%QSU	*r* = 0.241	*r* = 0.218	*r* = 0.352	*r* = −0.353
	(n.s.)	(n.s.)	(n.s.)	*p* < 0.001
% ID/mL spleen	*r* = 0.128	*r* = 0.020	*r* = 0.113	*r* = −0.094
	(n.s.)	(n.s.)	(n.s.)	(n.s.)
ICG R-15	*r* = −0.688	*r* = −0.521	–	–
	*p* < 0.001	*p* < 0.001		
PDR	*r* = −0.670	*r* = −0.547	–	–
	*p* < 0.001	*p* < 0.001		

Q-SPECT/CT, Quantitative single photon emission computed tomography; ICG, Indocyanine green; CTP, Child-Turcotte-Pugh; MELD, Model for end stage liver disease; LV, Liver Volume; %QLU, Percentage Quantitative Liver Uptake; %ID/mL, Injected Dose per milliliter; SV, Spleen Volume; %QSU, Percentage Quantitative Spleen Uptake; ICG R-15, Indocyanine Green retention rate at 15 min; PDR, Plasma Disappearance rate; #Pearson correlation (2-tailed) analysis.

### 3.4. Performance of Q-SPECT and ICG for disease severity and survival in DC

**Figure 1:**
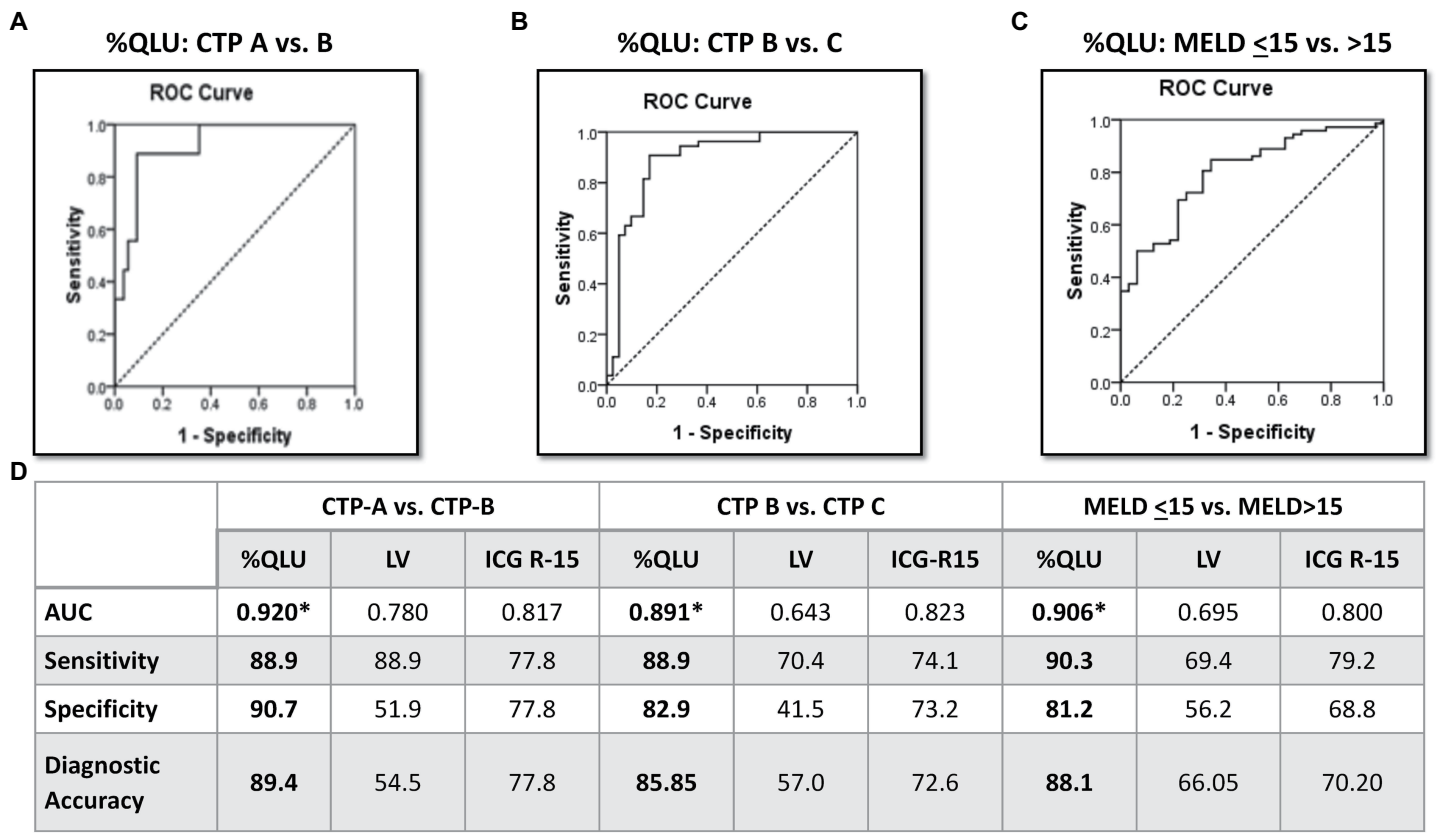
Diagnostic performance of Q-SPECT/CT and ICG-R15 for disease severity in cirrhosis Receiver operating characteristics for percentage quantitative liver uptake (%QLU) to discriminate **(A)** CTP-A vs. B, **(B)** CTP-B vs. C, and **(C)** MELD ≤15 vs. MELD>15 groups, **(D)** performance of %QLU, liver volume (LV), and indocyanine green retention (ICG-15) for CTP and MELD based prognostic categories.

**Figure 2:**
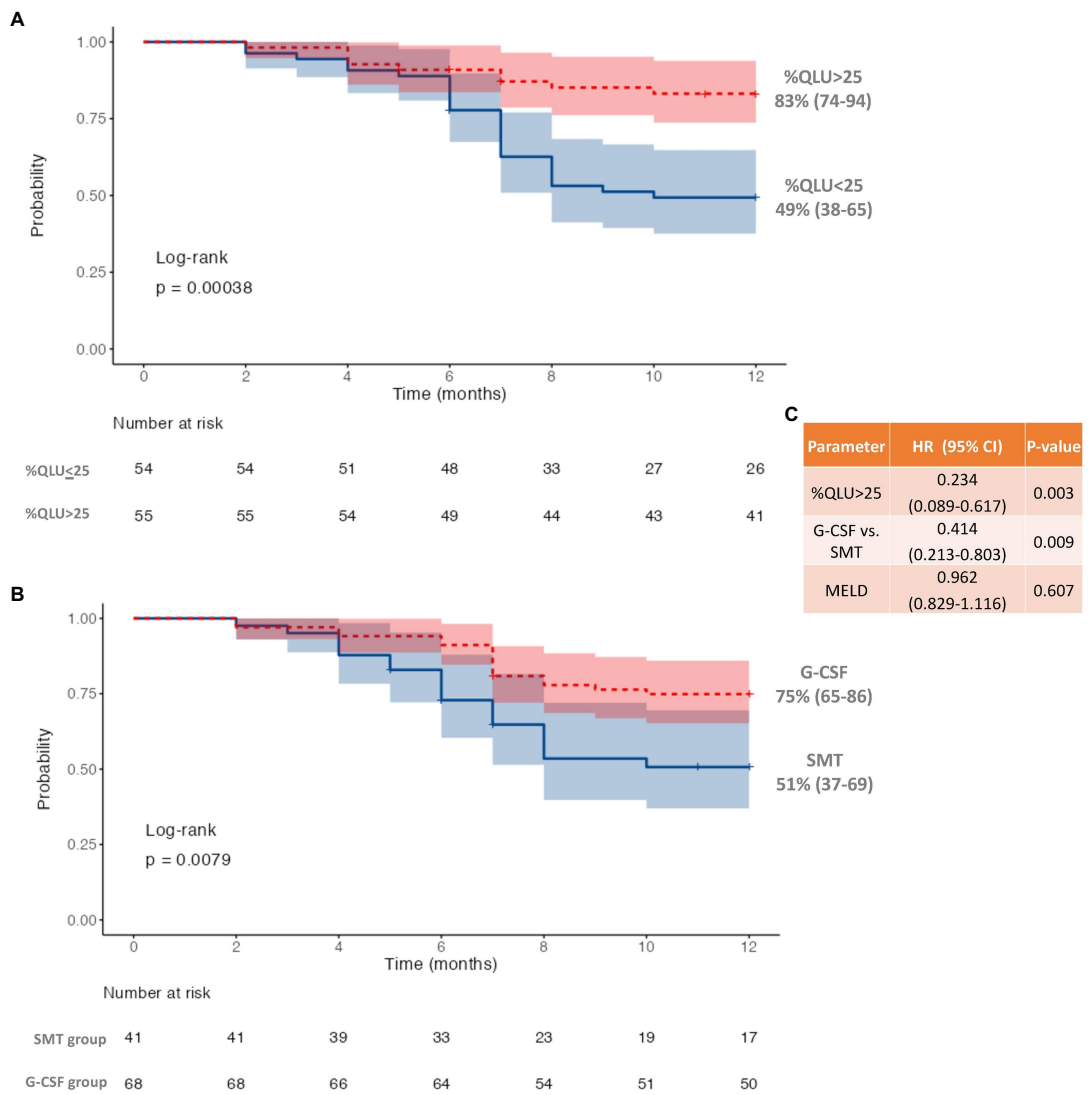
Twelve-month survival in decompensated cirrhosis **(A)** based on quantitative liver uptake (%QLU), **(B)** based on granulocyte colony-stimulating factor (G-CSF) treatment, and **(C)** Cox-regression for independent predictors of mortality.

On R.O.C. analysis (Figure 1), compared to ICG and Liver volume assessment, %QLU showed the highest area under the R.O.C. curve (AUC) for discriminating CTP-A from B (0.920), CTP-B from C (0.891), and MELD≤15 from MELD>15 groups (0.906). The %QLU above 37.2% could differentiate CTP-A from B cirrhosis with sensitivity and specificity of 88.9 and 90.7%. A value above 22.2% could discriminate CTP-B from C cirrhosis with sensitivity and specificity of 88.9 and 82.9%. A value above 18.7% could classify low MELD from high MELD patients with sensitivity and specificity of 90.3 and 81.2%. Patients with %QLU > 25 at baseline were associated with significantly better 12-month survival than those with %QLU ≤25 (83% vs. 49%, *p* < 0.001) (Figure 2A).

### 3.5. Impact of G-CSF on stem cells, severity, survival, and Q-SEPCT in cirrhosis

Following the first cycle of G-CSF treatment, there was a significant (p < 0.001) increase in %CD34+ cell count (0.8 to 16.4, *p* < 0.001) following G-CSF treatment on day 6 as compared to the “day 0” counts (Supplementary Table S3; Supplementary Figure S11). However, no significant rise was seen in the %CD 34+ cell count (0.85 to 0.90, *p* > 0.05) in the SMT group (Supplementary Table S3; Supplementary Figure S12). The 12-month survival was significantly better in the G-CSF group (75%) than in the SMT group (51%), *p* = 0.008 (Figure 2B). Better survival in G-CSF-treated patients was associated with a significant increase in % QLU at 12 months compared with the baseline (26.1 ± 10.6 to 31.2 ± 12.2, *p* = 0.001) (Table 3). While in group B, the %QLU at 12 months did not change from the baseline value. The observed liver SPECT findings corroborated a significant improvement in CTP and MELD scores only in the G-CSF group. No significant change was noted in any other parameter, including the ICG-R-15 value (*p* = 0.07) at 12 months versus the baseline value (Table 3).

**Table 3:** Change in disease severity scores, Q-SPECT/CT and ICG parameters in G-CSF treated (Group A) and Standard medical therapy groups (Group B).

Parameter	Group A			Group B		
	Baseline	Follow up	*p*-Value^#^	Baseline	Follow up	*p*-Value^#^
	Mean ± SD	Mean ± SD		Mean ± SD	Mean ± SD	
	Range	Range		Range	Range	
	Median	Median		Median	Median	
CTP	9.16 ± 1.65	7.40 ± 1.15	<0.001	8.14 ± 1.09	8.78 ± 1.88	0.264
	(6–13)	(5–10)		(6–10)	(7–12)	
	9	7		8	8	
MELD	13.61 ± 2.89	11.48 ± 2.63	<0.001	13.07 ± 2.73	14.85 ± 3.27	0.391
	(8–19)	(7–17)		(8–18)	(11–22)	
	14	12		13	14.5	
*Q-SPECT-liver parameters*						
%QLU	26.09 ± 10.6	31.21 ± 12.19	0.001	27.49 ± 9.3	26.4 ± 11.6	0.667
LV	976 ± 275	1,025 ± 251	0.215	955 ± 206	988 ± 260	0.691
% ID/mL (Liver)	0.028 ± 0.014	0.031 ± 0.010	0.165	0.029 ± 0.011	0.027 ± 0.007	0.484
*Q-SPECT-spleen parameters*						
%QSU	31.5 ± 12.67	29.10 ± 10.95	0.335	32.3 ± 12.4	30.7 ± 13.0	0.120
SV	674 ± 364	638 ± 340	0.552	486 ± 191	558 ± 222	0.213
% ID/mL (Spleen)	0.052 ± 0.020	0.050 ± 0.017	0.469	0.056 ± 0.019	0.049 ± 0.025	0.189
ICG R-15	46.7 ± 12.0	42.7 ± 12.6	0.066	43.09 ± 8.29	43.34 ± 10.36	0.814
PDR	5.93 ± 4.93	6.49 ± 4.18	0.148	5.73 ± 1.36	5.88 ± 1.54	0.937

Q-SPECT/CT, Quantitative single photon emission computed tomography; ICG, Indocyanine green; CTP, Child-Turcotte-Pugh; MELD, Model for end stage liver disease; LV, Liver Volume; %QLU, Percentage Quantitative Liver Uptake; %ID/mL, Injected Dose per milliliter; SV, Spleen Volume; %QSU, Percentage Quantitative Spleen Uptake; ICG R-15, Indocyanine Green retention rate at 15 min; PDR, Plasma Disappearance rate.

**Figure 3:**
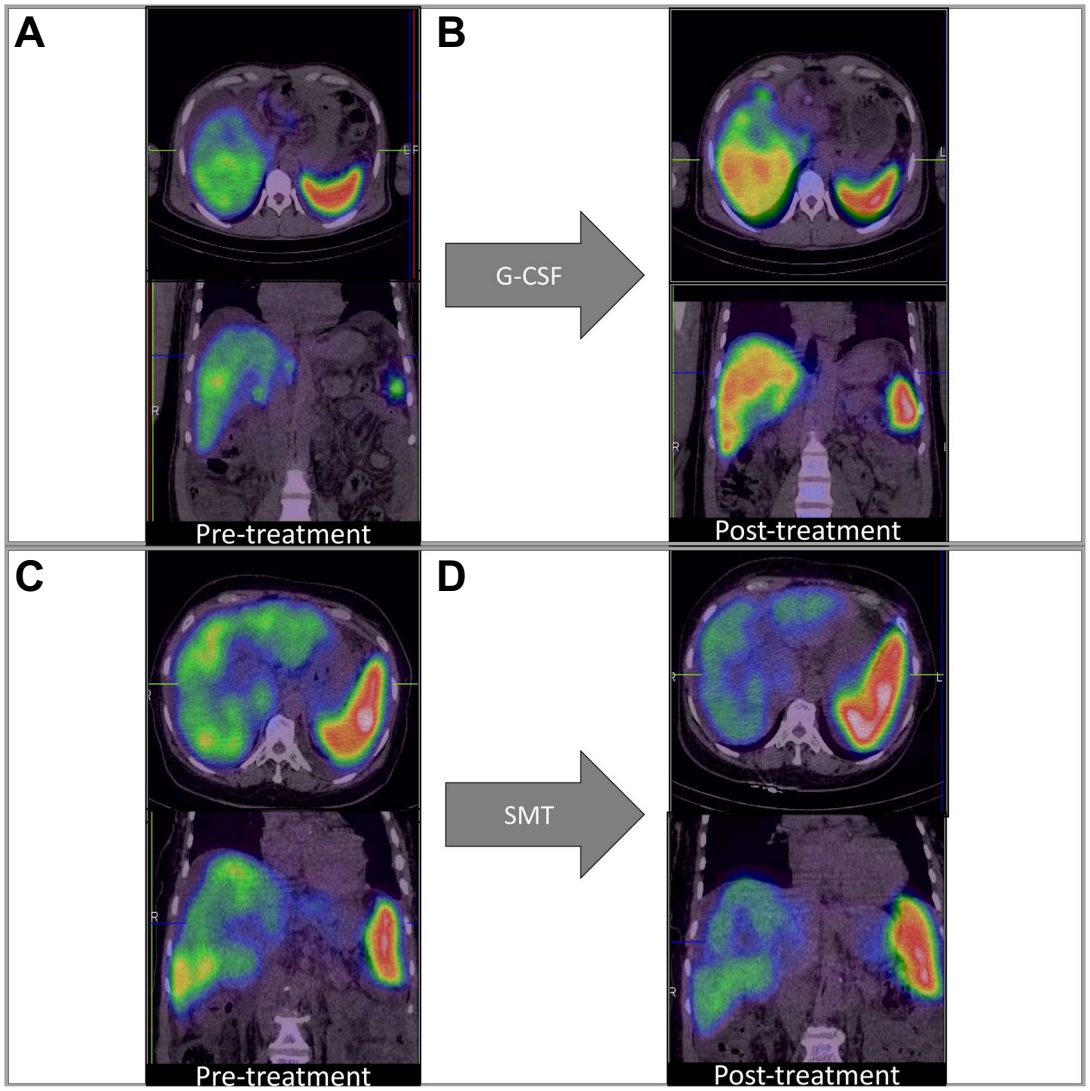
Q-SPECT/CT liver images with granulocyte colony-stimulating factor; G-CSF treatment [Panels **(A,B)**] and standard medical treatment (SMT) [Panels **(C,D)**] in representative DC patients. **(A,B)** Rise in %QLU (30 to 45%) and LV (964 to 1,016 cc) after G-CSF treatment **(C,D)** Fall in %QLU (41 to 36%) and LV (1,264 to 965 cc) after SMT treatment.

On adjusting for disease severity (MELD), baseline %QLU [H.R.: 0.234, 76.6% lower] and G-CSF treatment [H.R.: 0.414, 58.6% lower] were independently associated with a lower hazard of mortality in DC (Figure 2C). A representative ^99m^Tc-SC SPECT scan demonstrating an increase in %QLU and LV in a DC patient following G-CSF treatment at 12 months is shown in Figure 3A, while no change in the parameters was noted in a patient who received SMT (Figure 3B).

## 4. Discussion

We reported a novel non-invasive tool, i.e., Q-SPECT/CT, with parameters such as fractional liver uptake and liver volumes that exhibited a significant correlation with reference standard ICG test and clinical scores (CTP and MELD) in DC patients. %QLU showed the best discriminative ability for severity and survival in DC patients. Moreover, improvement in survival of patients with G-CSF correlated with improvement in %QLU values. Thus, advocating its use and further validation to assess the liver functions in DC patients semi-quantitatively. Our results are in agreement with Zuckerman et al. (3), who reported an inverse correlation (*r* = −0.64, *p* < 0.001) between %QLU and CTP scores. They also reported a negative correlation (*r* = −0.84, *p* < 0.001) between %QLU and ICG in patients with compensated cirrhosis. In another study, a significant correlation (*p* = 0.001) was reported between CTP class and ^99m^Tc-SC scan findings and was shown relevant for predicting prognosis in H.C.C. patients (16).

In liver cirrhosis, there is a high functional heterogeneity among the hepatic segments, which requires dynamic imaging that can estimate spatial functional distribution in the liver (16, 27–29). The “gold standard” ICG test lack such evaluation. Therefore, in the present study, we compared the diagnostic accuracy of ^99m^Tc-SC SPECT with the ICG test for estimating liver severity. On R.O.C. analysis, we demonstrated that %QLU on Q-SPECT/CT was a more accurate parameter than LV and ICG tests for stratifying DC patients according to their CTP and MELD scores.

In a recent study, the total liver function, which is a product of functional liver volume and liver/spleen counts, showed a sensitivity and specificity of 88.0 and 86.0% for differentiation of CTP A from B cirrhosis (16). In our study, the %QLU parameter offered a better sensitivity and specificity of 88.9 and 90.7% for classifying CTP-A from B cirrhosis. However, the ICG-R15 showed lower sensitivity and specificity (77.8% each) for such differentiation. The sub-optimal diagnostic performance of ICG in end-stage liver disease patients can be attributed to hyperbilirubinemia, intrahepatic shunt, or capillarization due to the same transport system of bile in hepatocytes, which could underestimate the ICG R-15 values in patients with DC (10–15). Therefore, the quantitative liver SPECT may add valuable information to DC patients.

G-CSF is a hematopoietic growth factor that stimulates the bone marrow to produce and release granulocytes and stem cells to the injured region (30–32). Further, the mobilization of CD34+ cells, triggered by G-CSF, may be helpful in hepatic tissue repair and regeneration (22, 24, 25). In the present study, we evaluated the diagnostic utility of quantitative liver SPECT for functional liver assessment and response evaluation to GCSF treatment in DC patients.

In two clinical studies, an improvement in one-year survival and disease severity score with multiple cycles of GCSF has been reported (25, 26). In another study, Garg et al. showed that 12 doses of G-CSF (5 μg/kg/dose) nearly doubled the survival in a group of ACLF patients predominantly of alcoholic etiology (22). It was observed that the %QLU at 12-mo post-G-CSF treatment was significantly higher (*p* = 0.002) than noted at the baseline level. These image findings thus suggest an improvement in liver function following G-CSF treatment.

Furthermore, we observed that the %QLU in G-CSF treated group of patients corroborated with the corresponding improvement in CTP and MELD score in group-A. However, both groups observed no significant change in ICG R-15 values. This finding may be attributed to intrahepatic shunts and capillarization found in cirrhosis which may falsely reflect the retention/clearance of the dye from the hepatocytes (33, 34). It is thus highlighted that the %QLU parameter of ^99m^Tc-SC-SPECT may be used non-invasively for accurate response evaluation to therapies in liver cirrhosis of different etiologies.

Limitations of the study include single-center design and limited generalization to outpatient DC patients; however, we could demonstrate the utility of Q-SPECT in a relatively large number of DC patients. We could not demonstrate the correlation of splenic SPECT parameters with CTP, MELD, or ICG R-15 indices, suggesting the limited utility of splenic parameters for evaluating liver functions. We hypothesize that splenic SPECT would reflect portal hypertension and its severity, which is poorly represented by ICG, CTP, or MELD scores and hence not reflected in our study.

In conclusion, we showed that Q-SPECT/CT of the liver is a reliable marker of quantitative liver functions in DC patients. %QLU correlates well with disease severity and reference standard (ICG test) in cirrhosis. %QLU can be used to non-invasively assess the severity, outcomes, and response to disease-modifying therapies such as G-CSF in cirrhosis.

## Lay summary

Quantitative liver function assessment is often challenging in liver cirrhosis patients. We developed and validated a novel single photon emission computed tomography-based fractional liver uptake parameter for non-invasive semi-quantitative evaluation of liver functions in cirrhosis. The fractional liver uptake correlated well with the gold standard; indocyanine green test, disease severity scores, and patient survival in cirrhosis. G-CSF treatment improved disease severity scores, survival, and fractional liver uptake, while no change was observed with standard medical therapy in decompensated cirrhosis.

## Data availability statement

The datasets presented in this article are not readily available because the data are not publicly available due to privacy or ethical restrictions. Requests to access the datasets should be directed to drbsingh5144@yahoo.com.

## Ethics statement

The studies involving human participants were reviewed and approved by PGIMER Institute Ethics Committee. The patients/participants provided their written informed consent to participate in this study.

## Author contributions

AKa, BS, and VS: conceptualization. AKa, NV, VS, and AD: data curation. AKa and NV: formal analysis, visualization, and writing – original draft. AKa, BS, VS, AKu, NV, AD, and SK: investigation. AKa, BS, VS, NV, AD, and RS: methodology. BS and VS: project administration and resources. AKa, NV, and AKu: software. BS and VS: supervision. BS and NV: validation. AKa, NV, and BS: writing – review and editing.

## Conflict of interest

The authors declare that the research was conducted in the absence of any commercial or financial relationships that could be construed as a potential conflict of interest.

## Publisher’s note

All claims expressed in this article are solely those of the authors and do not necessarily represent those of their affiliated organizations, or those of the publisher, the editors and the reviewers. Any product that may be evaluated in this article, or claim that may be made by its manufacturer, is not guaranteed or endorsed by the publisher.

## Abbreviations

^99m^Tc: Technetium-99 m
99mTc-SC: 99mTc-Sulfur colloid
AUC: Area Under the Curve
CTP: Child-Turcotte-Pugh
DC: Decompensated Cirrhosis
FACS: Fluorescence-activated cell sorting
G-CSF: Granulocyte-colony stimulating factor
HBV: Hepatitis B virus
HCV: Hepatitis C virus
ICG: Indocyanine Green
INR: International normalized ratio
LV: Liver volume
MELD: Model for end-stage liver disease
NASH: Non-alcoholic steatohepatitis
Q.L.U.: Quantitative Liver Uptake
Q.S.U.: Quantitative Spleen Uptake
R.C.P.: Radiochemical Purity
R.O.C.: Receiver Operator Characteristics
R.O.I.: Region of interest
SC: Sulfur-Colloid
SMT: Standard medical therapy
SPECT: Single Photon Emission Computed Tomography
S.V.: Spleen volume
