# Supplementary material for: Quantitative liver SPECT/CT is a novel tool to assess liver function, prognosis, and response to treatment in cirrhosis
Source: Front Med (Lausanne). 2023 Mar 22;10:1118531. doi: 10.3389/fmed.2023.1118531 (PMC10073445; doi:10.3389/fmed.2023.1118531)
Supplement: Supplementary file 1 [file Data_Sheet_1.DOCX]

**Quantitative liver SPECT/CT is a novel tool to assess liver function, prognosis, and response to treatment in cirrhosis**

**Supplementary Figures**

**Figure S1: Patient Recruitment**


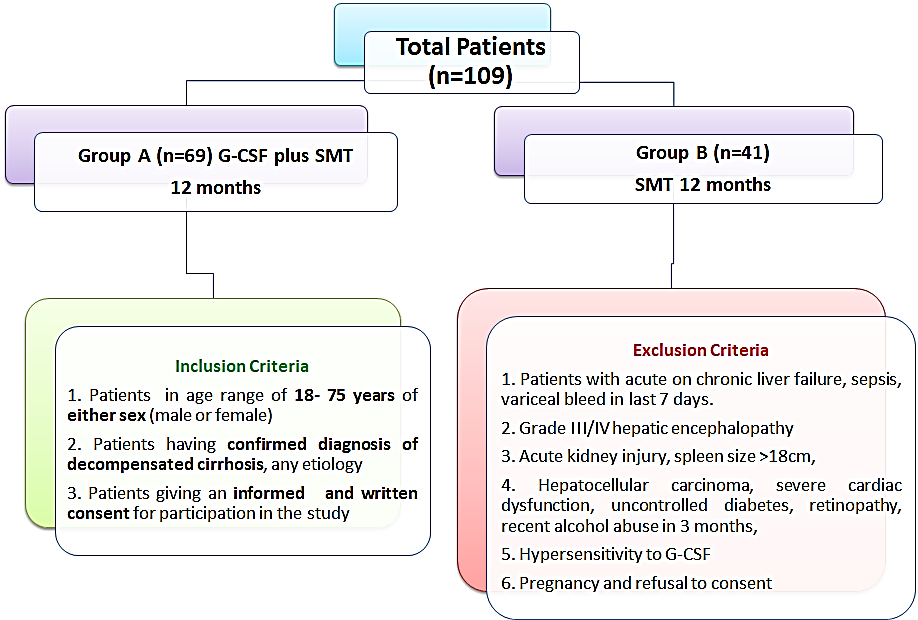


**Figure S2: Work Flow chart of the various investigations carried out at different time points in the study population**


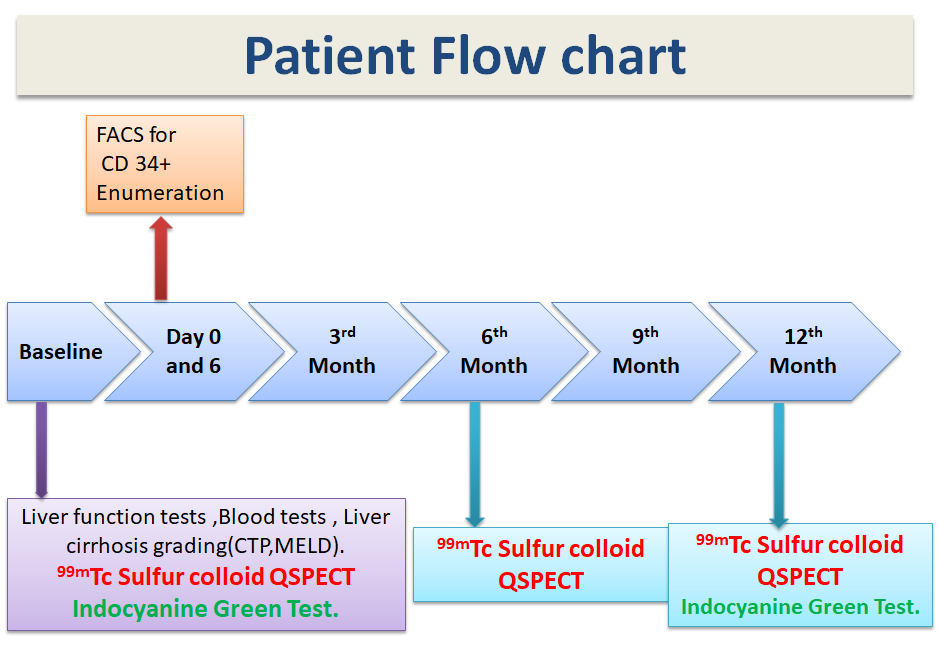


**Figure-S3A: Phantom Standardization: Jaszczak Phantom with various inserts and syringes used for filling with varying volumes of 6.0-30.0 mL of radioactivity of ^99m^Tc.**


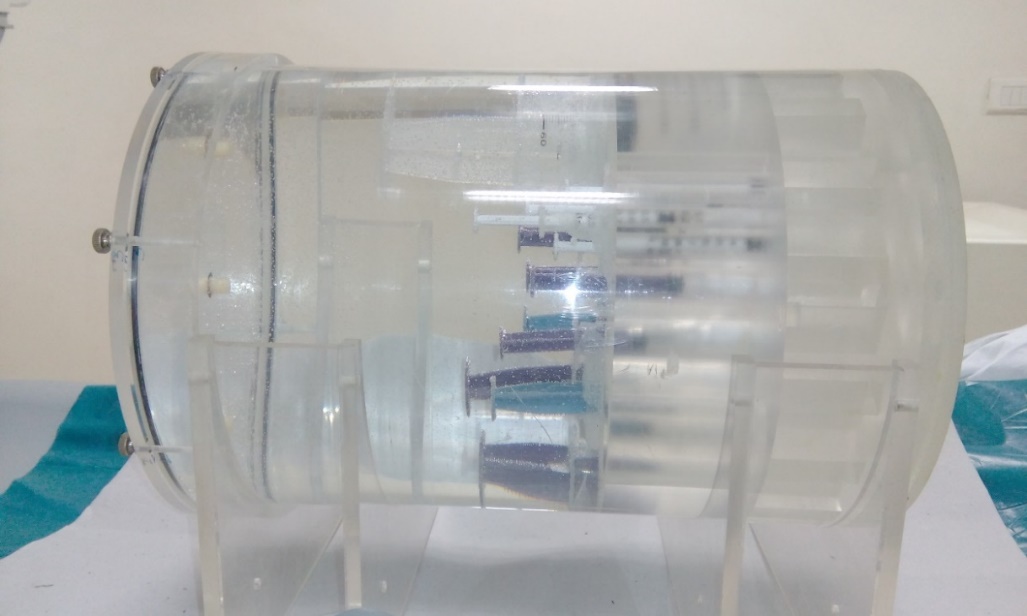


**Figure-S3B: Phantom Standardization: Jaszczak Phantom with various inserts and syringes used for filling with varying volumes of 500.0- 3200mL of radioactivity of ^99m^ Tc.**


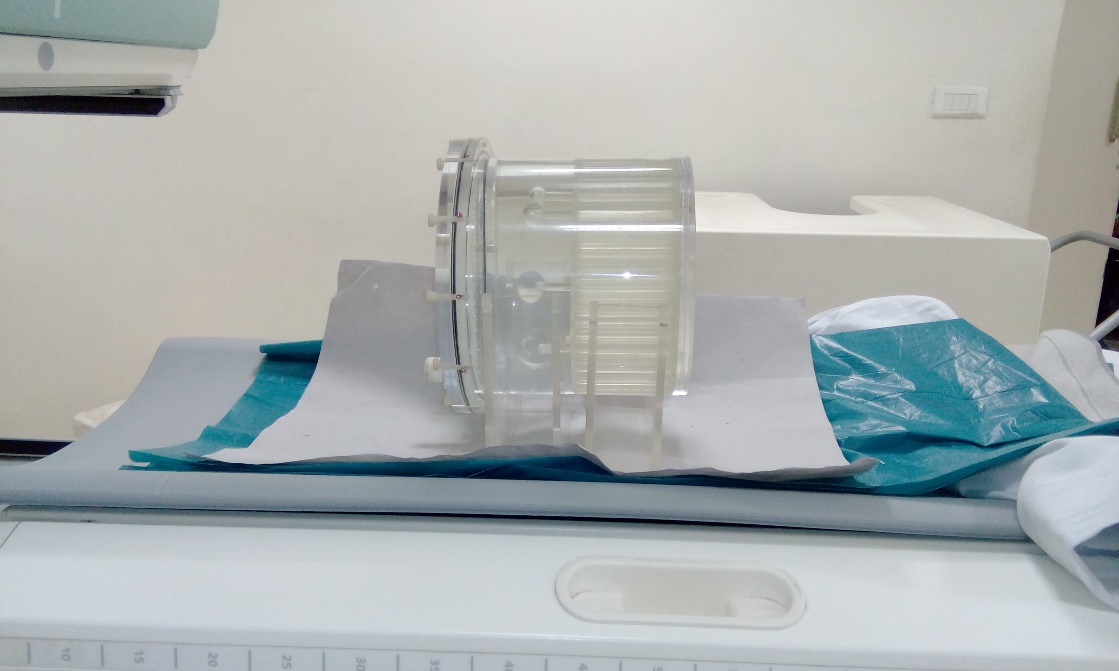


A series of phantom studies were performed to calculate the threshold value of the maximal pixel radioactivity by using varying radioactivity concentrations (0.77µCi/mL to 4.09 µCi/mL) and volumes (6.0 to 30 mL and 500-3200.0 mL). This method was used effectively to convert counts/voxel to µCi/mL. For all the phantom and patients’ studies, a dual headed gamma camera (Symbia- T16, SIEMENS, Germany) was used

**Figure-S4: Phantom Image reconstruction: Image of Jaszczak SPECT Phantom with the appropriate inserts used for threshold calculations.**


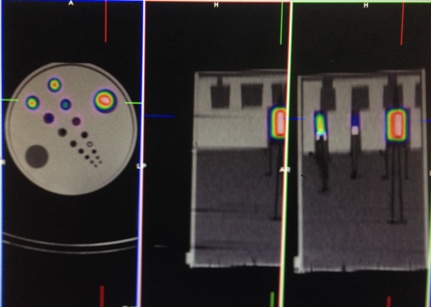


Phantom image acquisition (Single Photon emission computed tomography-SPECT) was done using a dual-headed gamma camera (Symbia- T16, SIEMENS, Erlangen, Germany) over 360^0^ circular orbit in 120 projections (20sec/projection) in 128 x 128 matrix with zoom factor of 1.0.

**Figure S5: Scatter plot of actual volume and SPECT measured volume as calculated by using attenuated corrected images using a threshold value of 38.0%.**

A highly significant (p<0.0001) correlation between the threshold value and the volume ranging from 500.0 mL to 3500.0 mL was 0.98 and was used to calculate the patients’ liver volume which most likely fall in this range.

**Figure S6: Scatter plot of SPECT measured concentration (counts/cc) versus actual concentration (µCi/cc) in attenuated corrected images using a threshold of 38.0%.**

Using the threshold of 38.0%, the SPECT reconstructed data was used to calculate the different range (0.77 µCi/mL to 4.09 µCi/mL) of radioactivity concentrations and compared with the actual concentrations used for this standardization procedure

**Figure S7: Patient Image Processing: results obtained after processing ^99m^Tc Sulfur colloid SPECT acquired patient data after standardization the processing protocol using phantom on 3 D volumetric analysis software.**


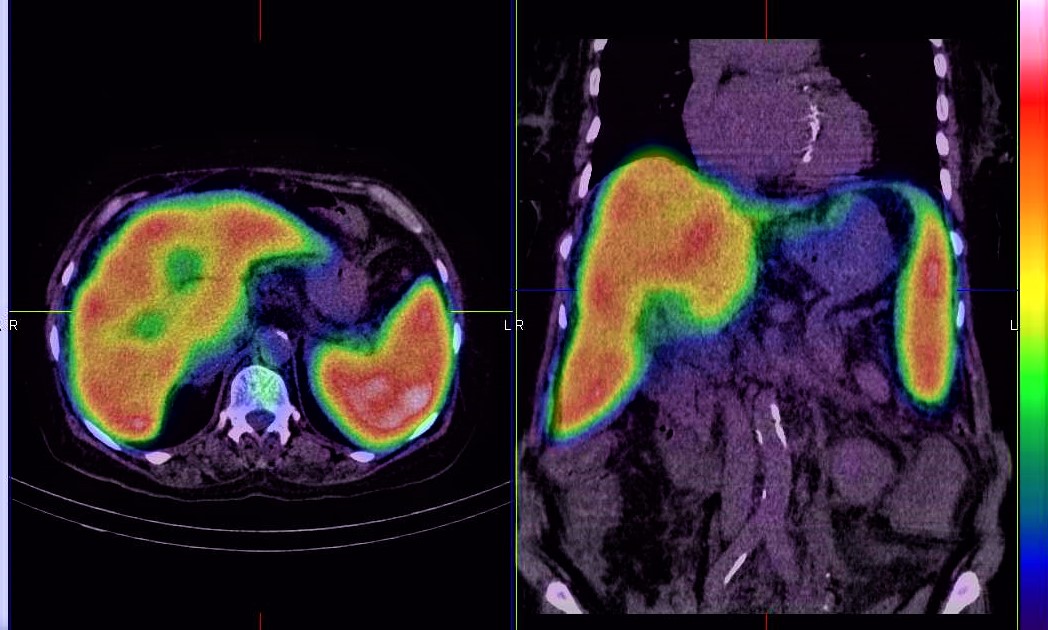

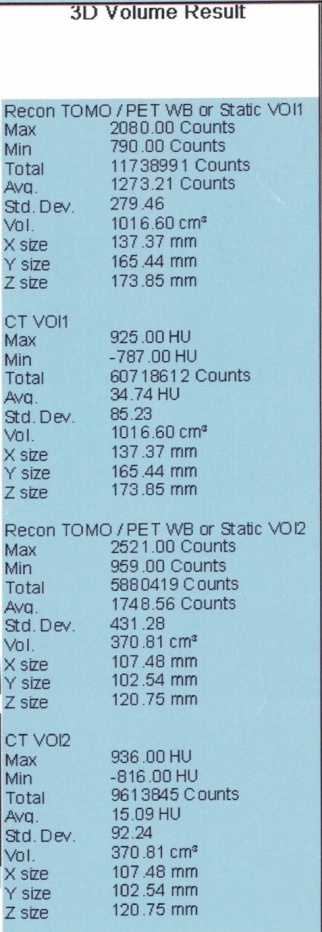


The best slice was identified to define the organ, and the region of interest (ROI) was drawn around the same. The ROI was checked for all the frames ensuring correct separation between the liver and spleen.

For volume measurements, the number of pixels containing activity greater than the threshold in all the checked sections was multiplied by the slice thickness and the values were summed-up and computed for all the patients.

For concentration/radioactivity measurements, the threshold value was subtracted from the values obtained for all the pixels in the ROI over the slices of interest. All the non-zero pixels with higher counts than the threshold value was used to calculate the radioactivity concentration. The output analysis of 3D volumetric Software 3.5.1, estimated total liver counts, total spleen counts, liver volume and spleen volume. All these output parameters were further used, and counts/voxels were converted into concentration units (µCi/mL) using the regression line obtained from the phantom measurements. The decay corrected percentage injected dose per mL of liver and spleen tissue (%ID/mL) was calculated. Liver and spleen uptake values were then obtained by multiplying the volume (mL) and %ID/mL.

**Figure S8 (A) Liver volume (LV) and (b) Quantitative liver uptake QLU (%) as a function of CTP scoring in CTP-A, CTP-B, CTP-C groups.**


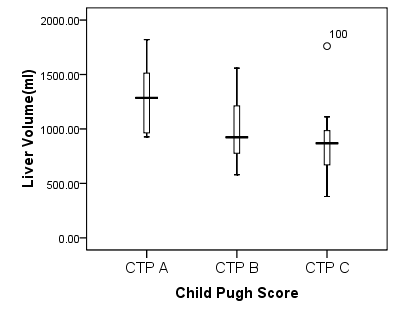


A


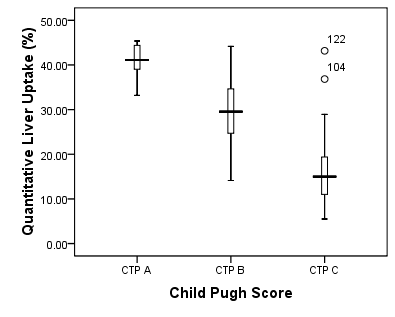


B

**SPECT parameters in CTP based categories:** The mean percent quantitative liver uptake (%-QLU) in the CTP-A, CTP-B and CTP-C groups were 41.20 ± 3.77, 29.80 ± 6.93 and 15.9 ± 7.83 respectively. The mean percent QLU value was highest in CTP-A group followed by CTP-B and CTP-C respectively. The value was significantly (p<0.001) higher in CTP-A than both in CTP-B and CTP-C groups. Further, the mean value in CTP-B was significantly (p<0.001) higher than in CTP-C. Box and Whisker graph demonstrating higher QLU (%) in CTP-A group than the other two (CTP-B, CTP-C) is presented in Figure-S8a. Similar findings were observed for liver volume (LV) estimates in the three CTP-based groups (Figure-S8b). The quantitative liver SPECT findings thus suggested that both %QLU and LV are found to be sensitive parameters for the differentiation of cirrhosis grading non-invasively under CTP based disease classification. On the other hand, the %ID/mL of liver did not differ amongst the three CTP patients’ groups.

**Figure S9: (A) Quantitative liver uptake (QLU) and (B) Liver volume (LV) as measured by SPECT of liver in two Model End Stage Liver Disease classes (MELD<15, MELD≥15)**


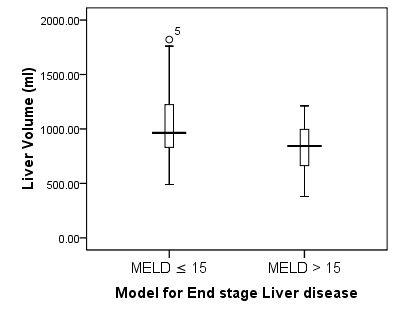


A


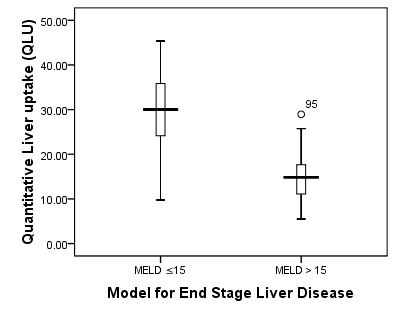


B

**Q-SEPCT in MELD based categories:** The mean ±SD values of LV were observed to be 1021±288 mL and 819±205 mL in MELD<15 (n=77) and MELD>15 (n=32) groups respectively. The %QLU values were 30.1 ± 9.0 and 15.25 ± 5.80 in the two MELD groups and were significantly higher (p<0.001) in MELD<15 group.

**Figure S10 - ICG- values in (a) three Child Pugh classes (CTP-A, CTP-B, CTP-C) of patients and (b) Model End Stage Liver Disease classes (MELD-I and MELD-II)**


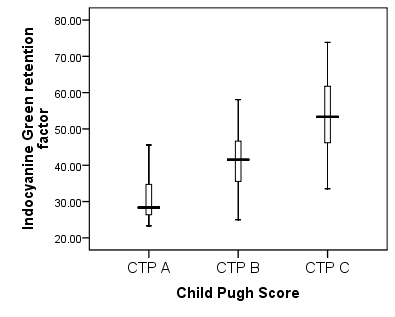


A


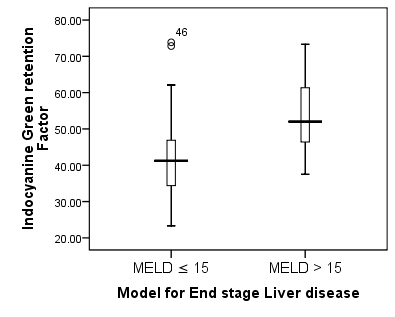


B

**ICG-test in CTP and MELD based categories:** ICG hepatic clearance test at baseline was performed in 104/109 patients. The ICG data was analyzed and quantitative parameters i.e., the ICG retention factor (ICG R-15) and plasma disappearance rate (PDR) were evaluated. The ICG R-15 values in CTP-A, CTP-B and CTP-C groups were 35.22 ± 8.33 %, 44.4 ± 10.50 % and 54.10 ± 12.03 % respectively. This indicates the retention of ICG at 15-min was lowest (p<0.001) in CTP-A group followed by CTP-B and CTP-C groups The ICG R-15 values of patients (n=72) in MELD-I group were significantly (p<0.001) lower (44.62 ± 12.08 %) than that (53.97 ± 10.71 %) observed in patients (n=32) of MELD-II group. The PDR was significantly (p<0.001) higher (5.59 ± 1.84) in MELD<15 (4.24 ± 1.39) than that seen in MELD>15 group of patients.

**Figure S11 :Fluorescence activated cell sorting to estimate the number of cell marker CD34^+^ cells (hematopoietic stem cells) in the peripheral blood samples pre and post G-CSF therapy day 0 (A) and day 6 (B) sample.**

**
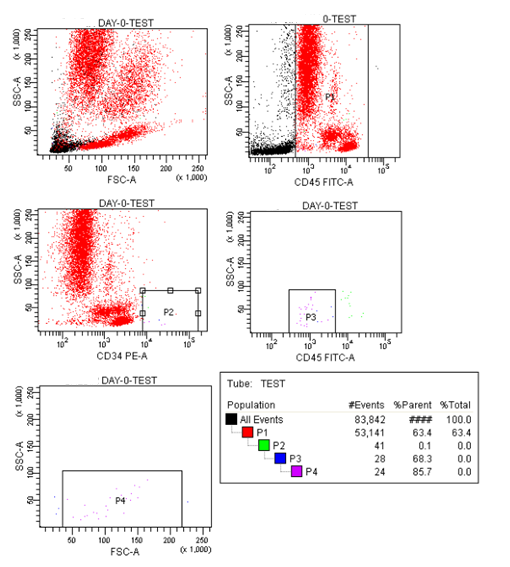
**

A

**
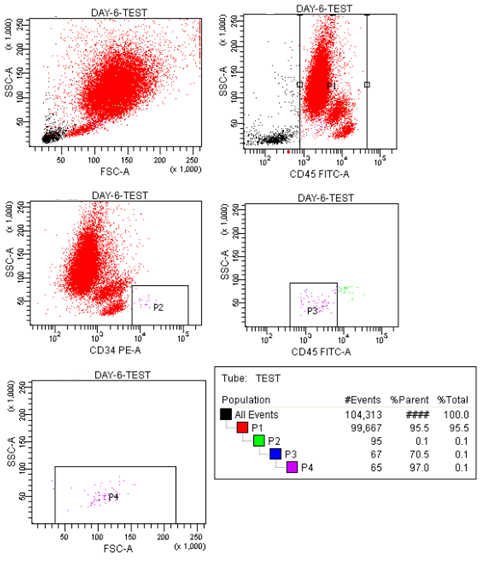
**

B

The FACS analysis demonstrating a sharp rise (spike) in CD34+ cells at day 6^th^ (in comparison with CD34+ cells’ number at day ‘0’) in the G-CSF treated patient.

**Figure S12 - Fluorescence activated cell sorting to estimate the number of cell markers CD34^+^ cells (hematopoietic stem cells) in the peripheral blood samples in standard medical therapy (group B) - day 0 (A) and day 6 (B) sample**

**
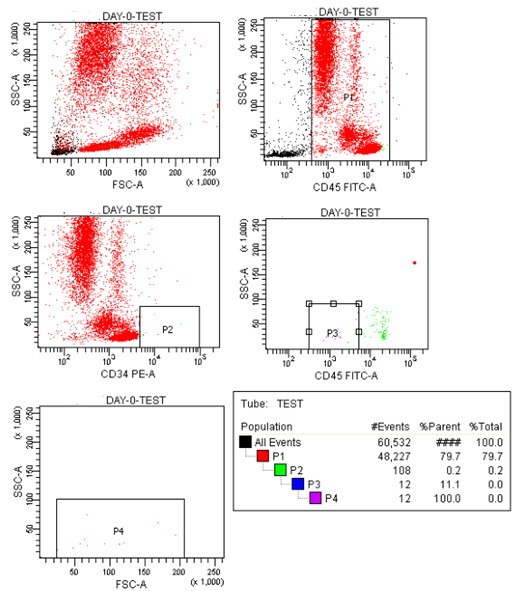
**

A

**
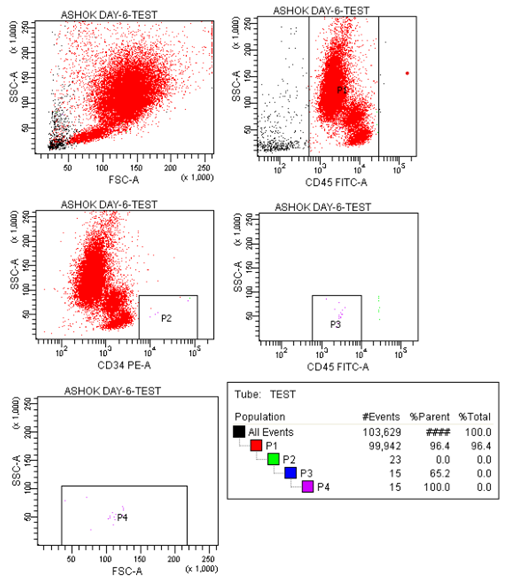
**

B

The FACS analysis demonstrating that he standard medical therapy (SMT) resulted in no change (no spike) in the CD 34+ cells at day 6 from baseline day 0.
