# Supplementary material for: Quantitative liver SPECT/CT is a novel tool to assess liver function, prognosis, and response to treatment in cirrhosis
Source: Front Med (Lausanne). 2023 Mar 22;10:1118531. doi: 10.3389/fmed.2023.1118531 (PMC10073445; doi:10.3389/fmed.2023.1118531)
Supplement: Supplementary file 2 [file Data_Sheet_2.DOCX]

**Quantitative liver SPECT/CT is a novel tool to assess liver function, prognosis, and response to treatment in cirrhosis**

**Supplementary Tables**

**Table S1: SPECT measured concentration (counts/cc) and actual concentration (µCi/cc) in attenuated corrected images using a threshold of 38.0%.**

| **Sr. No.** | **Phantom Concentration (µCi/cc)** | **SPECT measured concentration (counts/cc)** |
| --- | --- | --- |
| **1** | 0.779 | 4576.35 |
| **2** | 1.308 | 8812.45 |
| **3** | 2.00 | 13600.50 |
| **4** | 2.669 | 18331.38 |
| **5** | 3.244 | 19861.25 |
| **6** | 3.665 | 25026.18 |
| **7** | 4.095 | 27917.35 |

**Table S2: Baseline characteristics of study population**

| **Baseline Characteristics** | **Group-A (G-CSF)**  **(n=68)** | **Group-B (SMT)**  **(n=41)** | **p-value** |
| --- | --- | --- | --- |
| **Age** | 50.7±9.60 years | 52.85±9.8 years | 0.663 |
| **Gender** |  | | |
| **Males** | 58 (85.0%) | 37 (90.0%) | 0.434 |
| **Females** | 10 (15.0%) | 4 (10.0%) |  |
| **Disease (cirrhosis) Etiology** |  | | |
| **Alcoholic Liver Disease (ALD)** | 36 (52.9%) | 23 (56.0%) | 0.563 |
| **Non-Alcoholic Steatohepatitis (NASH)** | 14 (20.5%) | 6 (14.6% |  |
| **Hepatitis-C (HCV)** | 4 (5.8%) | 4 (9.7%) |  |
| **ALD + HCV** | 5 (7.3% | 2 (4.8%) |  |
| **Hepatitis-B (HBV)** | 4 (5.8%) | 3 (7.3% |  |
| **ALD + HBV** | 2 (2.9%) | 1 (2.4%) |  |
| **Autoimmune Hepatitis (AIH)** | 2 (2.9%) | 2 (4.8%) |  |
| **Wilson** | 1 (1.4%) | -- |  |
| **Na (mEq/L)** | 137 ± 6.3 | 134.5 ± 3.8 | 0.274 |
| **Serum Creatinine (mg/dL)** | 0.84 ± 0.22 | 0.94 ± 0.32 | 0.25 |
| **Bilirubin (mg/dL)** | 2.6 ± 1.55 | 2.07 ± 1.14 | 0.119 |
| **Albumin (g/dL)** | 3.19 ± 0.60 | 3.13 ± 0.68 | 0.368 |
| **INR** | 1.56 ± 0.30 | 1.46 ± 0.26 | 0.462 |
| **CTP-Median (Range)** | 9 (6-13) | 8 (6-13) | 0.519 |
| **MELD-Median (Range)** | 14 (8- 23) | 13 (7-23) | 0.688 |

G-CSF-Granulocyte Colony Stimulating factor, SMT-standard medical therapy, ALD- Alcoholic Liver Disease ,NASH- Non-Alcoholic Steatohepatitis , HCV - Hepatitis-C , HBV- Hepatitis-B , Na-Sodium, INR- International Normalized Ratio, CTP-Child Pugh Turcotte, MELD-Model for End Stage Liver Disease.

**Table S3: Pre and post treatment percent CD34+ cell counts (median values) in groups A and B patients.**

| **Group** | **Percent CD 34+ cells count (median value)** | | **p-value** |
| --- | --- | --- | --- |
|  | **At day 0**  **baseline** | **At day 6**  **post-treatment** |  |
| **Group-A (n=68)** | 0.8 (0.1-2.7) | 16.4 (2.8-64.23) | < 0.001 |
| **Group-B (n=41)** | 0.85 (0.1-2.3) | 0.90 (0.1-2.0) | 0.858 |
